# The Role of IRE-XBP1 Pathway in Regulation of Retinal Pigment Epithelium Tight Junctions

**DOI:** 10.1167/iovs.16-19232

**Published:** 2016-10

**Authors:** Jacey H. Ma, Joshua J. Wang, Junhua Li, Bruce A. Pfeffer, Yiming Zhong, Sarah X. Zhang

**Affiliations:** 1 Departments of Ophthalmology and Biochemistry, University at Buffalo, State University of New York, Buffalo, New York, United States; 2 SUNY Eye Institute, State University of New York, New York, United States; 3 Aier School of Ophthalmology, Central South University, Changsha, Hunan, China; 4 Research Service, Veterans Administration Western New York Healthcare System, Buffalo, New York, United States; 5 State Key Laboratory of Ophthalmology, Zhongshan Ophthalmic Center, Sun Yat-sen University, Guangzhou, Guangdong, China

**Keywords:** retinal pigment epithelium, tight junction, X-box binding protein 1

## Abstract

**Purpose:**

The retinal pigment epithelium (RPE) tight junctions play a pivotal role in maintaining the homeostatic environment of the neural retina. Herein, we investigated the role of X-box binding protein 1 (XBP1), an endoplasmic reticulum (ER) stress-responsive transcription factor, in regulation of RPE tight junctions.

**Methods:**

Human RPE cell line (ARPE-19) and primary primate RPE cells were used for in vitro experiments and RPE-specific XBP1 knockout (KO) mice were used for in vivo study. Endoplasmic reticulum stress was induced by a sublethal dose of thapsigargin or tunicamycin. XBP1 activation was manipulated by IRE inhibitor 4μ8C, which suppresses XBP1 mRNA splicing. The integrity of tight junctions and the involvement of calcium-dependent RhoA/Rho kinase pathway were examined.

**Results:**

Induction of ER stress by thapsigargin, but not tunicamycin, disrupted RPE tight junctions in ARPE-19 cells. Inhibition of XBP1 activation by 4μ8C resulted in a remarkable downregulation of tight junction proteins (ZO-1 and occludin) and defects in tight junction formation in the presence or absence of ER stress inducers. Overexpression of active XBP1 partially reversed 4μ8C-induced anomalies in tight junctions. Mechanistically, XBP1 inhibition resulted in increased intracellular Ca^2+^ concentration, upregulation of RhoA expression, redistribution of F-actin, and tight junction damage, which was attenuated by Rho kinase inhibitor Y27632. In vivo, deletion of XBP1 in the RPE resulted in defective RPE tight junctions accompanied by increased VEGF expression.

**Conclusions:**

Taken together, these results suggest a protective role of XBP1 in maintaining RPE tight junctions possibly through regulation of calcium-dependent RhoA/Rho kinase signaling and actin cytoskeletal reorganization.

The endoplasmic reticulum (ER) is the central organelle responsible for protein biosynthesis and posttranslational modification and maturation. These complex processes are dynamically regulated and tightly controlled by specific signaling pathways known as the unfolded protein response (UPR)^1^ to ensure the fidelity of protein production and maintain protein homeostasis. The activation of the UPR is mediated by three transmembrane proteins, namely inositol-requiring protein-1 (IRE1), activating transcription factor-6 (ATF6), and protein kinase RNA (PKR)-like ER kinase (PERK). In resting cells, these proteins are kept in an inactive state by binding to ER chaperone glucose-regulated protein 78 (GRP78, also known as immunoglobulin binding protein, BiP). During ER stress, where unfolded or misfolded proteins accumulate in the ER, GRP78 is sequestered by the unfolded/misfolded proteins, and the dissociation from its above mentioned binding partners results in the activation of ER stress sensors and their downstream UPR signaling pathways. Apart from protein folding, the ER also serves as the major storage site for intracellular calcium ion, a universal signaling messenger governing a diverse range of cellular processes such as cell migration and muscle contraction. While depletion of calcium causes ER stress and activates the UPR, failure to restore ER homeostasis by the UPR often leads to aberrant calcium release from the ER resulting in cell injury.^2,3^ In endothelial cells, excessive cytosolic calcium causes disassociation of tight junctions and increases vascular permeability.^4,5^

The retinal pigment epithelium (RPE) is a single layer of cuboidal cells that are essential for maintaining the normal structure and function of photoreceptors. Loss of functional RPE is implicated as an early and critical event in the development of AMD.^6^ In addition, adjacent RPE cells form tight junctions, which act as a selective barrier between the fenestrated choroidal capillaries and the neural retina, also known as the outer blood–retinal barrier (BRB). Defects in RPE junctions contribute to the pathologic processes of choroidal neovascularization (CNV) in wet AMD and retinal edema in diabetic retinopathy (DR).^7,8^ While the exact molecular mechanisms in controlling the formation and function of RPE tight junctions remain elusive, emerging evidence suggests a potential role of ER stress in the regulation of tight junction proteins and RPE cells.^9–11^ Recently, we demonstrated that exposing RPE cells to cigarette smoke extract, a potent inducer of ER stress,^12–14^ resulted in severe structural and functional damage to tight junctions associated with dysregulated F-actin cytoskeleton.^12^ In contrast, overexpression of ERp29, an ER chaperone, significantly increases the expression of tight junction proteins and improves the barrier function of RPE cells.^12^ These findings indicate that the ER stress-activated UPR pathways are possibly involved in the regulation of RPE tight junctions.

The IRE1-X-box binding protein 1 (XBP1) pathway is the most conserved core branch of the UPR, playing a pivotal role in maintaining the ER homeostasis.^15,16^ The active form of XBP1, spliced XBP1 (XBP1s), is a major transcription factor that regulates a subset of ER chaperones and ER-associated degradation (ERAD) genes during ER stress.^17,18^ Insufficient activation of XBP1 renders stressed cells susceptible to apoptosis^17^ and augments inflammation in retinal blood vessels.^19^ In addition, XBP1 is required for maintaining the basal level of intracellular calcium. Pancreatic α-cells lacking XBP1 gene exhibit significantly higher level of calcium accompanied by impaired glucagon secretion.^20^ In RPE cells, activation of XBP1 promotes cell survival by reducing ER stress and inducing antioxidant genes.^13,14,17^ However, the role of IRE1-XBP1 pathway in RPE tight junction formation remains unknown. In the current study, we investigated the implication of this pathway in regulation of tight junction proteins in the RPE and explored the potential mechanisms underlying ER stress-induced tight junction changes.

## Materials and Methods

### Animals

All animal procedures were approved by the Institutional Animal Care and Use Committees at the University at Buffalo, State University of New York, and in accordance with the ARVO Statement for the Use of Animals in Ophthalmic and Vision Research. Generation of RPE-specific XBP1 knockout mice was described elsewhere.^21^ Cre negative, XBP1 floxed littermates were used as control. To induce diabetes, 8-week-old mice were given five consecutive intraperitoneal injections of streptozotocin (STZ, 50 mg/kg/d; Sigma-Aldrich Corp., St. Louis, MO, USA) or vehicle as control. Four weeks after STZ injection, mice were euthanized and eyes were harvested for analyses.

### Cell Culture

A human RPE cell line (ARPE-19)^22^ was obtained from American Type Culture Collection (ATCC, Manassas, VA, USA) and maintained in DMEM/F12 50/50 Mix (Mediatech, Inc., Manassas, VA, USA) supplied with 10% (vol/vol) fetal bovine serum (FBS; Gibco Invitrogen Corporation, Carlsbad, CA, USA). When cells reached 90% confluence, the serum concentration in the culture medium was gradually reduced to complete deprivation. Fully confluent cells were then maintained in serum-free culture medium supplemented with Insulin-Transferrin-selenium-A (Gibco) providing a final concentration of insulin 10 mg/L, transferin 5.5 mg/L, and sodium selenite 6.7 ug/L, 0.5% (wt/vol) bovine serum albumin (BSA; Calboichem, La Jolla, CA, USA), and 1 mM sodium pyruvate (Mediatech, Inc.) for 2 to 3 additional weeks to enhance differentiation and promote the formation of tight junction complexes. Differentiated cells were treated with tunicamycin (Tm, 1 μg/ml), thapsigargin (Tg, 1 μM; Sigma-Aldrich Corp.), or 4μ8C^23^ (David Ron, MD, Cambridge University, and EMD Millipore, Temecula, CA, USA) at doses and time as specified in the Results section.

Rhesus macaque RPE (MRPE) cells^24^ (fourth passage, derived from primary cultures prepared in accordance with IACUC and ARVO guidelines) were seeded in 4-well Falcon CultureSlides (BD Falcon, Bedford, MA, USA) that had been coated with mouse laminin (Sigma-Aldrich Corp.) or polyornithine (Sigma-Aldrich Corp.). As described previously,^24^ MRPE cells were cultured in low-calcium medium ([Ca^2+^] below 0.1 mM) for proliferation. Upon confluence, the cultures were switched to medium containing [Ca^2+^] at 0.5 mM, and maintained for a period of up to 3 months for differentiation. The complete formulation of the final MRPE culture medium is provided in Supplemental Materials and Methods. The monolayers of MRPE cells exhibited a differentiated morphology, including development of melanin pigmentation, phase-bright polygonal cell packing, and dome formation and were used for subsequent experimental treatments.

### Transduction of Adenoviruses in ARPE-19 Cells

Differentiated ARPE-19 cells in 6-well culture plates were transduced with adenoviruses expressing spliced XBP1 at MOI of 20 as described previously.^13,14^ Adenoviruses expressing LacZ were used as control. After 24 hours of transduction, cells were subjected to desired treatment.

### Western Blot Analysis

Radioimmuno precipitation assay (RIPA) buffer with protease inhibitor mixture, PMSF, and sodium orthovanadate (Santa Cruz Biotechnology, Santa Cruz, CA, USA) was used to extract the proteins from cells or tissues. A BCA protein assay kit (Thermo Fisher Scientific, Inc., Rockford, IL, USA) was used to measure protein concentration. Twenty-five micrograms of protein were resolved by SDS-PAGE and blotted with specific antibodies: anti-XBP1, anti-ATF4 (CREB2; Santa Cruz Biotechnology); anti-cleaved caspase-3, anti-ZO-1, anti-occludin (Invitrogen, Carlsbad, CA, USA), anti-p-eIF2α, anti-CHOP, anti-p58^IPK^ (Cell Signaling Technology, Boston, MA, USA); or anti-KDEL, anti-ATF6 (Abcam, Cambridge, MA, USA). The same membrane was stripped and reblotted with an anti-β-actin antibody (Abcam) as loading control. After incubation with peroxidase-labeled secondary antibodies (Vector Laboratories, Inc., Burlingame, CA, USA), membranes were developed with SuperSignal West Dura Chemiluminescent Substrate (Thermo Fisher Scientific, Inc., Rockford, IL, USA). Protein bands were quantified by densitometry, normalized to β-actin (loading control).

### Immunofluorescence Staining and Morphologic Study of Tight Junctions

ARPE-19 or MRPE cells were fixed in 4% paraformaldehyde for 10 minutes and permeabilized with 0.3% Triton X-100 in PBS for 10 minutes. After blocking with 3% BSA for 1 hour, cells were incubated with rabbit anti-ZO-1 or mouse anti-occludin antibodies (Invitrogen) overnight at 4°C. Then, cells were incubated with Alexa Fluor 488 goat anti-mouse, Alexa Fluor 488 goat anti-rabbit, or Texas red goat anti-rabbit antibodies (1:200, Molecular Probes; Invitrogen) for 1 hour. In some experiments, cytoskeleton was labeled with Alexa Fluor 594 conjugated phalloidin (1:200; Invitrogen). Cell nuclei were stained with 4′,6-diamidino-2-phenylindole (DAPI)-containing VECTASHIELD Antifade Mounting Medium (Vector Laboratories, Inc.) and digitally photographed using a Zeiss LSM confocal microscope (Carl Zeiss, Jena, Germany). To analyze the tight junction morphology, Z-scans were performed with a 0.41-μm interval from the apical to basal levels of the RPE cells, enabling a comprehensive analysis of three-dimensional orientation of tight junctions and cytoskeleton. Z-stack projection was performed and full resolution images were exported using Zeiss LSM Image Examiner software.

For mouse RPE staining, the eyeballs were fixed with 4% paraformaldehyde in PBS for 45 minutes and the retinas were carefully dissected out. The resulting eyecup were washed in PBS, blocked in 10% goat serum with 0.5% Triton X-100, and then incubated with rabbit anti-ZO-1 antibody (1:50; Invitrogen) overnight at 4°C. After incubation with secondary antibody, eyecups were treated with Alexa Fluor 594 - phalloidin (1:200; Invitrogen), flat mounted, and examined by confocal microscopy as above.

### Real-Time qPCR and RT-PCR

Total RNA from cultured cells or eyecups was extracted using TRIzol reagent (Invitrogen). The cDNA were synthesized using Maxima First Strand cDNA Synthesis Kits for RT-qPCR (Thermo Fisher Scientific, Inc.). Real-time RT-PCR was performed using SYBR Green PCR Master Mix (Bio-Rad Laboratories, Hercules, CA USA). Specific primers used for real-time PCR are as follows: human ZO-1 (forward) 5′-CAG CAA CTT TCA GAC CAC CA-3′ and (reverse) 5′- GTG CAG TTT CAC TTG GCA GA-3′; mouse VEGF (forward) 5′-GAC TTG TGT TGG GAG GAG GA-3′and (reverse) 5′-TCT GGA AGT GAG CCA ATG TG-3′. The levels of target genes were normalized by mouse 18S ribosomal RNA. Human XBP1 primers, (forward) 5′-CCA TGG ATT CTG GCG GTA TTG ACT −3′ and (reverse) 5′- CCA CAT TAG CTT GGC TCT CTG TCT −3′, were used to detect the unspliced and spliced XBP1 by RT-PCR.

### RhoA Activation Assay

RhoA activation was evaluated using RhoA Activation Assay kit (Cat # STA-403-A; Cell Biolabs, Inc., San Diego, CA, USA) following manufacturer's instruction. The assay utilizes Rhotekin Rho-binding domain (RBD) Agarose beads to pull down the active form of RhoA from cell lysate. Briefly, supernatant from cell lysate was incubated with RBD-beads for 1 hour. Beads were collected by centrifugation, washed, and resuspended in SDS-PAGE sample buffer. Activated RhoA was detected by western blot analysis.

### Intracellular Calcium Concentration [Ca^2+^]_i_ Measurement

Measurement of [Ca^2+^]_i_ was performed using the Ca^2+^-sensitive fluorescent dye Fluo-4/AM (Invitrogen). Briefly, monolayers of ARPE-19 cells in glass bottom culture dishes were loaded with Fluo-4/AM (5 μm) in phenol red-free DMEM/F12 with 0.5% BSA for 30 minutes. After washing, the cells were equilibrated with a dye-free DMEM/F12 (0.25% BSA) for at least 10 minutes. Life images were taken upon a Zeiss Axio Observer inverted microscope with excitation at 488 nm. The images were recorded every 20 second for at least 3 minutes. The fluorescent intensity was quantified by Zeiss Axio Observer software.

### Statistical Analysis

Statistical analyses were performed using unpaired Student's *t*-test when comparing two groups and 1-way ANOVA with Newman-Keuls multiple comparison test for three groups or more. The quantitative data were expressed as mean ± SD. Statistical differences were considered significant at a *P* value of less than 0.05.

## Results

### Inhibition of XBP1 Activation by a Small Molecule Inhibitor 4μ8C in RPE Cells

**Figure 1:**
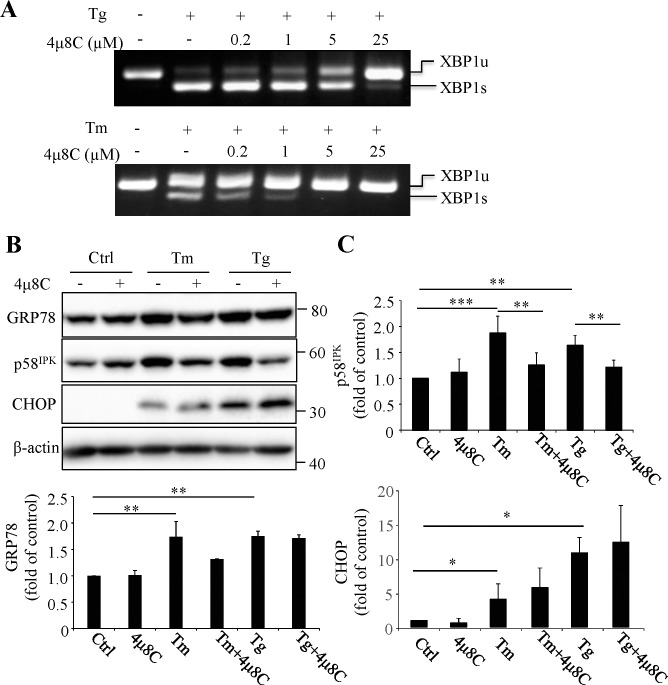
Inhibition of XBP1 activation by 4μ8C in ARPE-19 cells. (**A**) RT-PCR showing dose-dependent blockade of splicing of XBP1 mRNA by 4μ8C in ARPE-19 cells treated with 1 μM thapsigargin (Tg) or 1 μg/ml tunicamycin (Tm) for 6 hours. (**B**, **C**) ARPE19 cells were pretreated with 25 μM 4μ8C for 45 minutes followed by treatment with 1 μg/ml Tm or 1 μM Tg for 24 hours. (**B**) Representative images of Western blots of ER stress marker. (**C**) Protein levels of p58^IPK^, GRP78, and CHOP were quantified by densitometry and normalized to β-actin. Data represent means ± SD from three independent experiments, *P < 0.05, **P < 0.01, ***P < 0.001.

4μ8C is a small molecule inhibitor of IRE1α that specifically binds to the RNase domain of p-IRE1α, thereby inhibiting IRE1-mediated XBP1 mRNA splicing without affecting its kinase activity.^23^ We first tested the efficacy of 4μ8C inhibition of XBP1 activation in differentiated human ARPE-19 cells challenged with a sublethal dose of ER stress inducers thapsigargin or tunicamycin. As shown in Figure 1A, pretreatment of cells with 4μ8C dose-dependently inhibited the splicing of XBP1 mRNA induced by thapsigargin (Tg, 1 μM, 6 hours) or tunicamycin (Tm, 1 μg/ml, 6 hours). At concentration of 25 μM, 4μ8C completely blocked XBP1 mRNA splicing induced by Tg. Accordingly, this dose was chosen to be used to inhibit XBP1 activation in most experiments of this study. Next, we determined the effect of XBP1 inhibition on the activation of UPR pathways in ARPE-19 cells in the presence or absence of ER stress. In cells without challenge by ER stress inducers, 4μ8C did not change the protein expression levels of GRP78, p58^IPK^, CHOP (Figs. 1B, 1C), p-eIF2α, ATF4, or cleaved ATF6 (data not shown). Treatment with 1 μM Tg or 1 μg/ml Tm for 24 hours induced a significant increase in GRP78, p58^IPK^ and CHOP, indicating an induction of ER stress. Interestingly, no significant increase was observed in phosphorylation of eIF2α or ATF6 cleavage at this time point. Pretreatment with 25 μM 4μ8C for 45 minutes partially reduced the increase in GRP78 and p58^IPK^ protein levels but did not alter CHOP expression under ER stress conditions (Figs. 1B, 1C). These results suggest that inhibition of XBP1 activation attenuates ER stress-induced upregulation of ER chaperones but has no significant effect on UPR activation in differentiated RPE cells.

### Inhibition of XBP1 Activation Altered the Distribution and Expression of Tight Junction Proteins in RPE Cells

**Figure 2:**
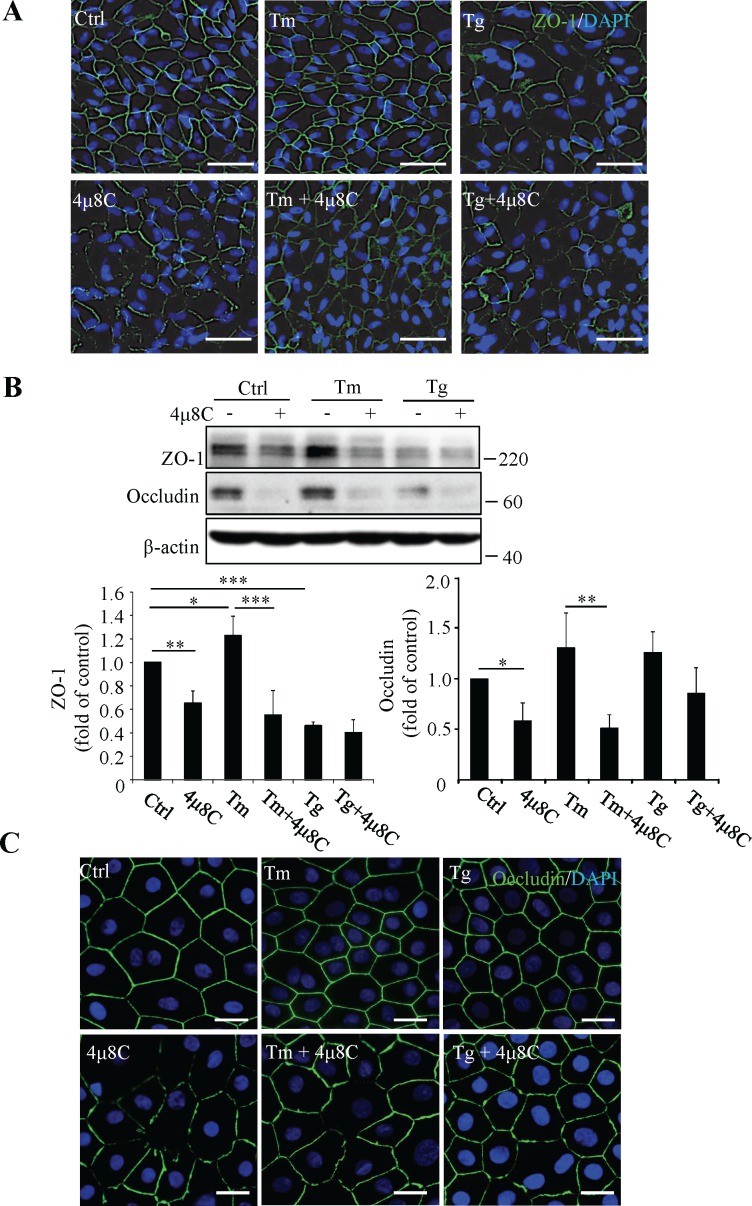
Effects of ER stress and XBP1 inhibition on tight junctions in cultured RPE cells. (**A**, **B**) Differentiated ARPE-19 cells were pretreated with 25 μM 4μ8C for 45 minutes followed by Tm (1 μg/ml) or Tg (1 μM) treatment for 24 hours. (**A**) Immunofluorescence staining of ZO-1. *Scale bars*: 50 μm. (**B**) Levels of tight junction proteins were examined by Western blot analysis and quantified by densitometry. Data are shown as means ± SD (*n* = 4), *P < 0.05, **P < 0.01, ***P < 0.001. (**C**) Primary MRPE cells were of pretreated with 25 μM 4μ8C for 45 minutes followed by Tm (1 μg/ml) or Tg (1 μM) treatment for 24 hours. Tight junction formation was evaluated by immunofluorescence staining of occluding. *Scale bars*: 20 μm.

To evaluate the role of ER stress and XBP1 activation in regulation of RPE tight junctions, we treated RPE cells with 25 μM 4μ8C for 45 minutes and subsequently with Tg (1 μM) or Tm (1 μg/ml) for 24 hours. Retinal pigment epithelial tight junctions were assessed by immunofluorescent labeling of ZO-1 (Fig. 2A). Our result showed that treatment with the SERCA inhibitor Tg, which depletes the calcium from the ER,^25^ resulted in a marked disruption of tight junctions in RPE cells, as evidenced by the discontinuities in, and reduced intensities of, the fluorescent signal routinely observed at cell borders in the control sample field. In contrast, treatment with Tm, an inhibitor of N-linked glycosylation, had no effect, compared with control treatment, on the labeling pattern of RPE tight junctions. Interestingly, cells pretreated with 4μ8C demonstrated defective tight junctions and reduced ZO-1 expression at the cell surface, which was exacerbated by Tm treatment (Fig. 2A). Western blot analysis confirmed that the protein levels of ZO-1 and occludin were significantly reduced in cells pretreated with 4μ8C in the presence or absence of Tm (Fig. 2B). However, inhibition of XBP1 by 4μ8C had no further effect on thapsigargin-induced ZO-1 loss. To further validate the effect of XBP1 inhibition on RPE tight junctions, we employed well-differentiated, normal diploid nonhuman primate RPE (MRPE) cells.^24^ As shown in Figure 2C, these cells demonstrate optimal hexagonal morphology and excellent tight junction formation revealed by immunofluorescent labeling of occludin. Intriguingly, neither Tm nor Tg resulted in any damage to tight junctions in MRPE cells, indicating that these cells are likely more resistant to ER stress. Conversely, cells pretreated with 4μ8C (25 μM) demonstrated reduced and irregularly distributed occludin regardless of the presence of ER stress inducer Tm or Tg (Fig. 2C). In addition, 4μ8C treatment for 24 hours significantly reduced the transepithelial electrical resistance (TEER) of ARPE-19 cells (Supplementary Fig. S1), but did not induce cell apoptosis or alter cell viability (Supplementary Figs. S2, S3). These results suggest that XBP1 activation may play a role in ER stress-related RPE barrier dysfunction.

### Overexpression of XBP1s Partially Reversed the Disruption of Tight Junction in ARPE19 Cells

**Figure 3:**
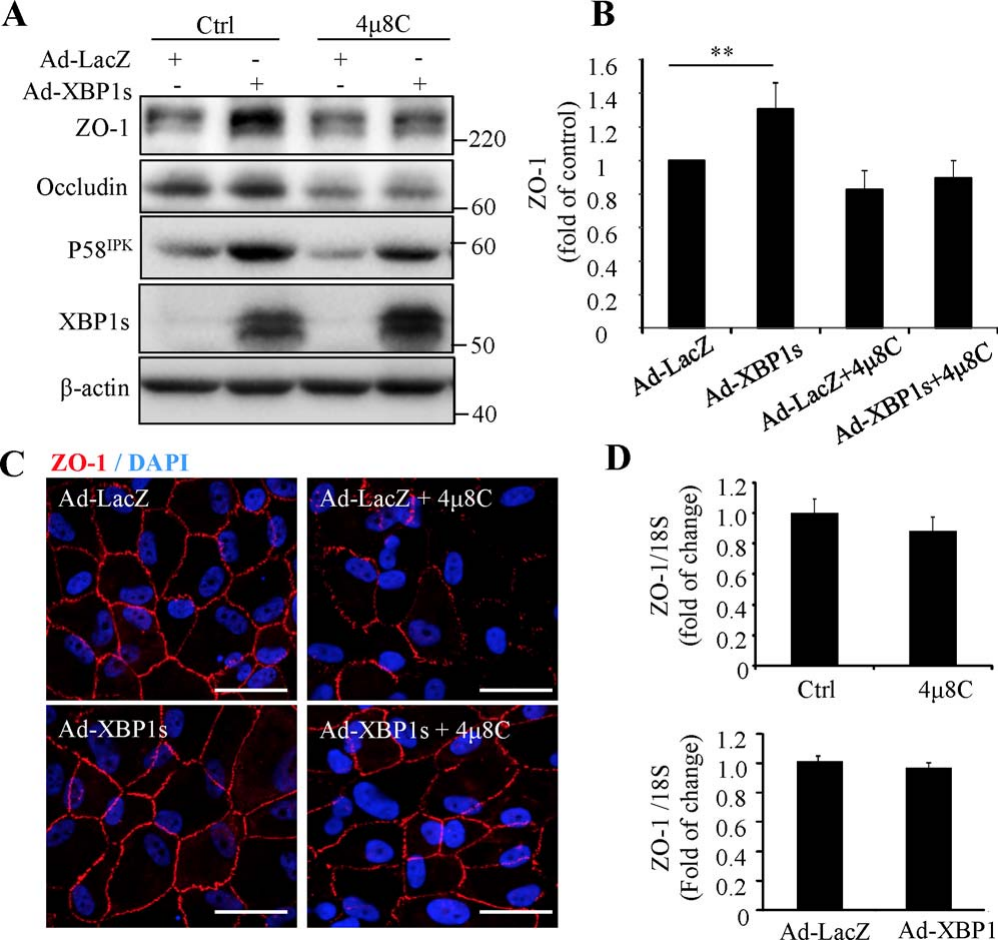
Overexpression of XBP1s increases ZO-1 expression and partially reverses the disruption of tight junction in ARPE-19 cells. Cells were transduced with Ad-XBP1s or control adenovirus (Ad-LacZ) for 24 hours and then treated with 25 μM 4μ8C for additional 24 hours. (**A**) Western blot analysis of tight junction proteins, XBP1s and p58^IPK^. (**B**) The protein level of ZO-1 was quantified by densitometry. Data are shown as means ± SD (*n* = 3), **P < 0.01. (**C**) Immunofluorescence staining for ZO-1 (*red*). Nuclei were stained with DAPI (*blue*). *Scale bars*: 50 μm. (**D**) mRNA levels of ZO-1 were assessed by real-time qPCR. Data are shown as means ± SD (*n* = 3).

To further evaluate the protective role of XBP1 in RPE tight junctions, we overexpressed XBP1s by adenovirus in ARPE-19 cells. After viral transduction, the cells were treated with 25 μM 4μ8C for 24 hours. The efficiency of adenoviral transduction was confirmed by high expression levels of XBP1s and its downstream target p58^IPK^ (Fig. 3A). Overexpression of XBP1s significantly enhanced the expression of ZO-1 (Figs. 3A, 3B) and largely prevented the disruption of tight junctions caused by 4μ8C (Fig. 3C). To examine whether XBP1s regulates ZO-1 transcription, the mRNA level of ZO-1 was measured by real-time PCR. Results show that neither XBP1 inhibition nor XBP1 overexpression altered the mRNA expression of ZO-1 (Fig. 3D), suggesting the effect of XBP1s on ZO-1 may occur at a posttranslational level.

### RhoA/Rho Kinase Pathway Signaling Was Involved in 4μ8C-Induced ZO-1 Perturbation

**Figure 4:**
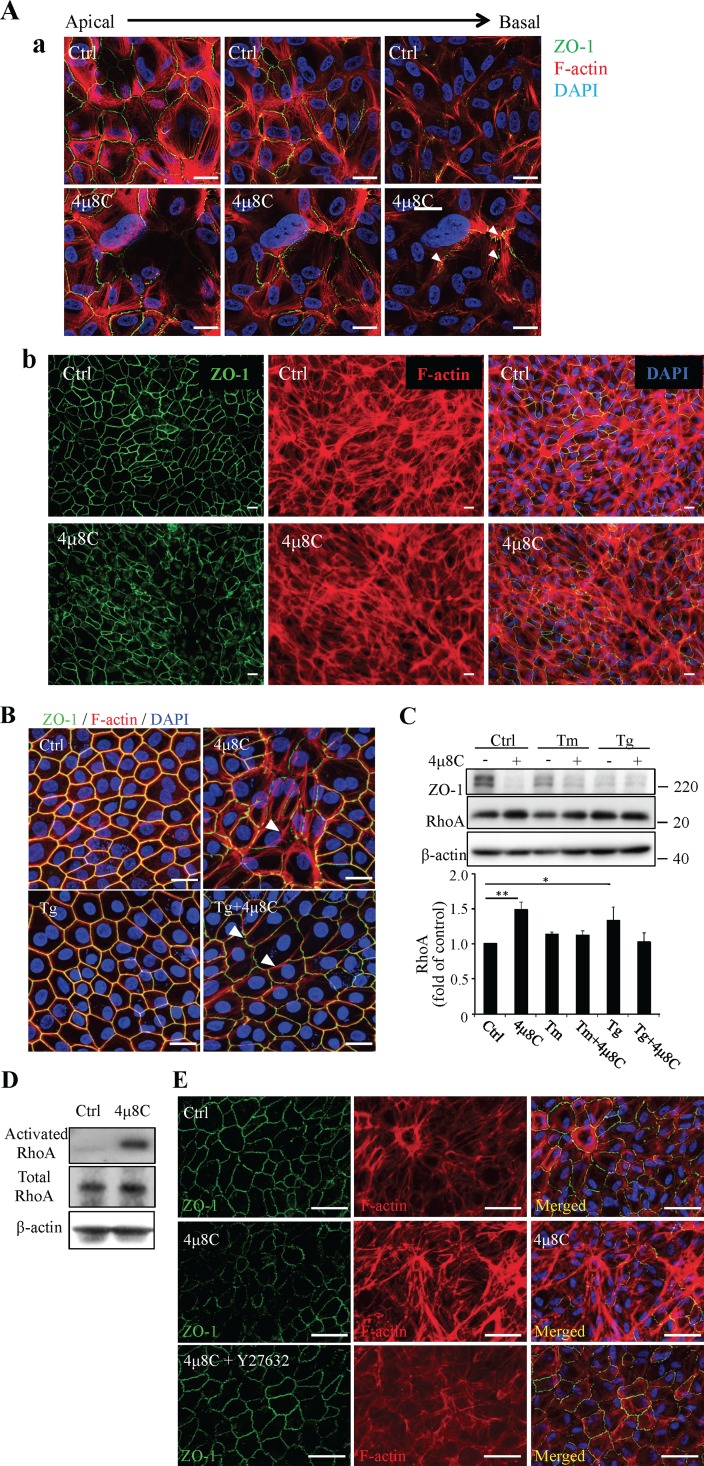
Effects of XBP1 inhibition on actin cytoskeletal organization and RhoA/Rho kinase signaling. (**A**) ARPE-19 cells were treated with 25 μM 4μ8C for 24 hours. Tight junction and actin cytoskeleton were labeled with immunostaining of ZO-1 (*green*) and phalloidin (*red*). (**a**) Representative confocal scans from apical to basal compartments. *Scale bars*: 50 μm. *Arrowheads* show colocalization of redistributed ZO-1 and actin filaments. (**b**) Images with low magnification. *Scale bars*: 20 μm. (**B**) Rhesus macaque RPE cells were treated with 4μ8C for 45 minutes prior to Tg treatment (1 μM, 24 hours). Double staining of ZO-1 (*green*) and phalloidin (*red*) show aberrant distribution of ZO-1 and cytoskeleton in 4μ8C-treated cells. *Arrowheads* show colocalization of redistributed ZO-1 and actin filaments. *Scale bars*: 20 μm. (**C**) ARPE19 cells were pretreated with 25 μM 4μ8C for 45 minutes and followed by Tm (1 μg/ml) or Tg (1 μM) treatment for 24 hours. Expression of RhoA was examined by western blot analysis and quantified by densitometry (means ± SD, *n* = 3, *P < 0.05, **P < 0.01). (**D**) RhoA activation was evaluated in ARPE-19 cells treated with 4μ8C for 24 hours using RhoA activation assay kit as described in Methods section. Results represent two independent experiments. (**E**) ARPE19 cells were pretreated with 1 μM Y27632 followed with 4μ8C (25 μM) treatment for 24 hours. Immunofluorescence staining of ZO-1 (*green*) and phalloidin (*red*) was performed to examine tight junctions and actin cytoskeleton. *Scale bars*: 50 μm. Images represent three independent experiments.

Previous studies have shown that activation of the RhoA/Rho kinase pathway leads to cytoskeleton rearrangement and consequent tight junction damage in the RPE.^26^ Herein, we investigated if the RhoA/Rho kinase pathway is implicated in RPE tight junction regulation by XBP1. First, we performed a double labeling of ZO-1 (with a monospecific antibody and fluor-conjugated secondary antibody) and cytoskeleton (with fluor-conjugated phalloidin) in RPE cells to identify the tight junctions and actin filaments, respectively. In control ARPE-19 cells, most actin filaments are circumferentially oriented at the level of the apical pole of the cells (Fig. 4A). In contrast, in cells treated with 4μ8C the actin filaments exhibited a more vertical orientation, with redistribution toward the cells' basal side. These redistributed actin filaments were colocalized with ZO-1immunoreactivity (arrowheads, Fig. 4A). Similar changes of actin cytoskeleton were observed in MRPE cells (Fig. 4B). Moreover, we found that XBP1 inhibition by 4μ8C or depletion of the ER calcium by Tg induced a significant increase in RhoA expression (Fig. 4C) and enhanced RhoA activity (Fig. 4D). To further confirm a role of the RhoA/Rho kinase pathway in tight junction damage, we pretreated ARPE-19 cells with specific Rho kinase inhibitor Y27632 (1 μM) for 45 minutes prior to 4μ8C treatment. We found that blockade of Rho kinase activity largely rescued the disruption of tight junctions and rearrangement of actin filaments induced by 4μ8C (Fig. 4E).

### XBP1 Inhibition Increases Intracellular Calcium Concentration in RPE Cells

**Figure 5:**
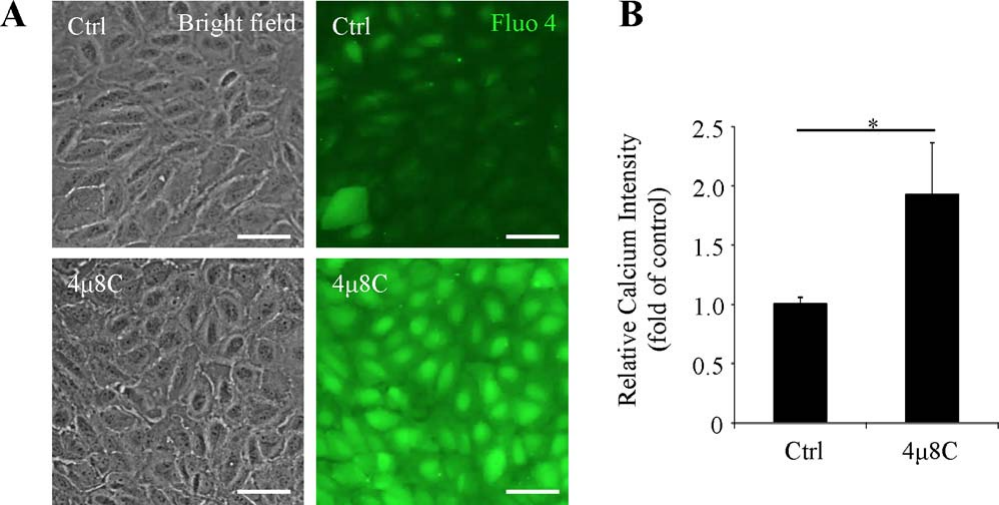
Inhibition of XBP1 activation increases intracellular calcium concentration. ARPE-19 cells were treated with 25 μM 4μ8C for 24 hours. Intracellular calcium concentrations were measured by Fluo-4/AM. (**A**) Representative bright field images (*left panel*) and Fluo-4/AM staining images (*right panel*). *Scale bars*: 50 μm. (**B**) Intracellular calcium concentration was evaluated by fluorescence intensity. Results represent mean ± SD of three independent experiments.

Intracellular calcium concentration is a critical factor in controlling cell junctions and permeability in epithelial cells.^27–29^ To evaluate if the changes of actin filaments and tight junctions are associated with calcium, we measured the intracellular calcium concentration ([Ca^2+^]_i_) by fluorescent probe Fluo-4/AM in ARPE-19 cells treated with or without 4μ8C (25 μM, 24 hours). We found that inhibition of XBP1 by 4μ8C increased [Ca^2+^]_i_ by almost 2-fold (Figs. 5A, 5B). This result suggests that XBP1 inhibition may lead to cytoskeleton rearrangement and tight junction damage through disruption of calcium homeostasis (see also Discussion, below).

### Conditional Knockout of XBP1 Results in Impaired Tight Junction Complex in Mouse RPE

**Figure 6:**
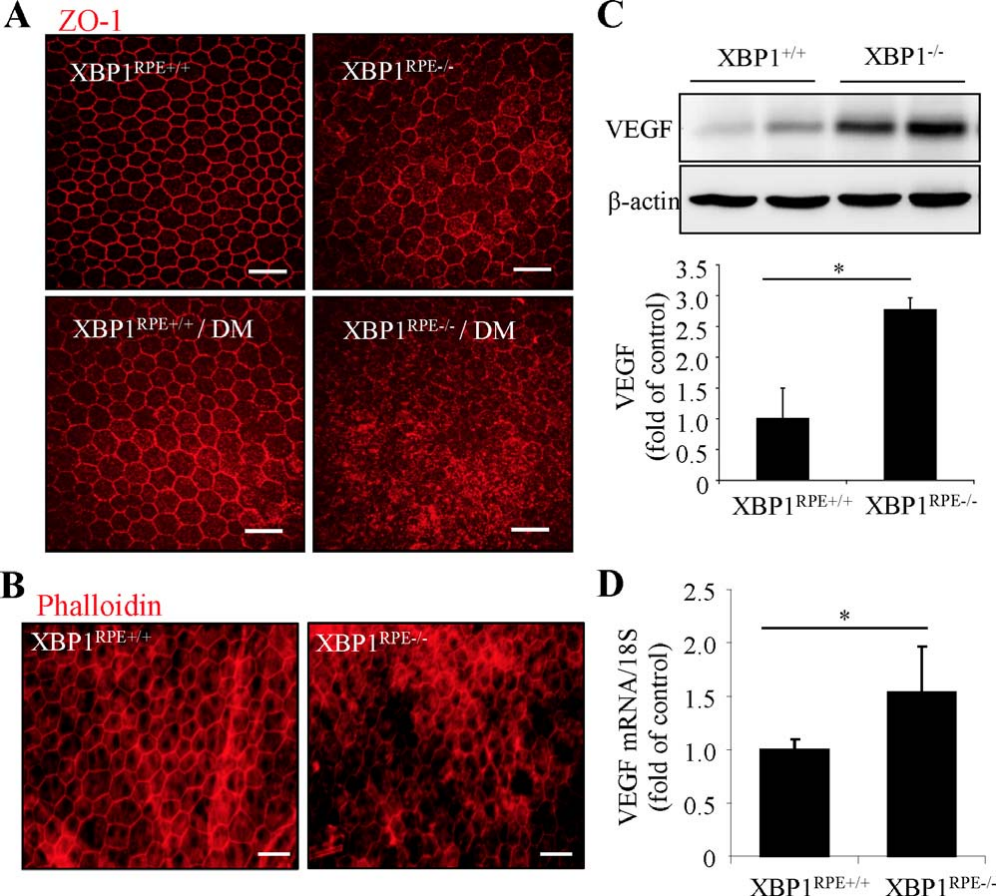
Loss of XBP1 results in defective RPE tight junctions and increases VEGF expression in vivo. (**A**, **B**) Diabetes was induced by STZ in RPE-specific XBP1 knockout (XBP1^RPE−/−^) or control (XBP1^RPE+/+^) mice. Retinal pigment epithelial wholemounts were stained with ZO-1 (**A**) or phalloidin (**B**). *Scale bars*: 50 μm. Images represent results from three mice in each group. (**C**) Protein levels of VEGF in mouse eyecups were analyzed by Western blot analysis and quantified by densitometry (mean ± SD, *n* = 6, *P < 0.05). (**D**) mRNA levels of VEGF in moue eyecups were determined by real-time qPCR. Results are presented as mean ± SD from 4 XBP1^RPE+/+^ mice and 6 XBP1^RPE−/−^ mice.

Retinal pigment epithelium–specific XBP1 knockout (XBP1^RPE−/−^) mice were generated in our previous study and induced diabetes by streptozotocin. To further confirm the role of endogenous XBP1 on RPE tight junctions, we examined the tight junctions by immunofluorescent labeling of ZO-1 (Fig. 6A) or using fluor-conjugated phalloidin (Fig. 6B) on RPE whole mounts from XBP1^RPE−/−^ and control mice with or without diabetes. We found morphologically altered RPE tight junctions in XBP1^RPE−/−^ mice compared with controls, and the observed defects in tight junction labeling patterns were intensified in diabetic conditions (Figs. 6A, 6B). Because increased VEGF expression has been shown to be a major factor inducing RPE tight junction dysfunction,^30^ we measured VEGF expression in mouse eyecups containing choroid and RPE isolated from XBP1^RPE−/−^ and control mice. We found that both mRNA and protein levels of VEGF were significantly increased in the eyecups of XBP1^RPE−/−^ mice (Figs. 6C, 6D). These results suggest a potential role of VEGF in mediating loss of tight junction functional integrity associated with XBP1 deficiency in RPE in vivo.

## Discussion

**Figure 7:**
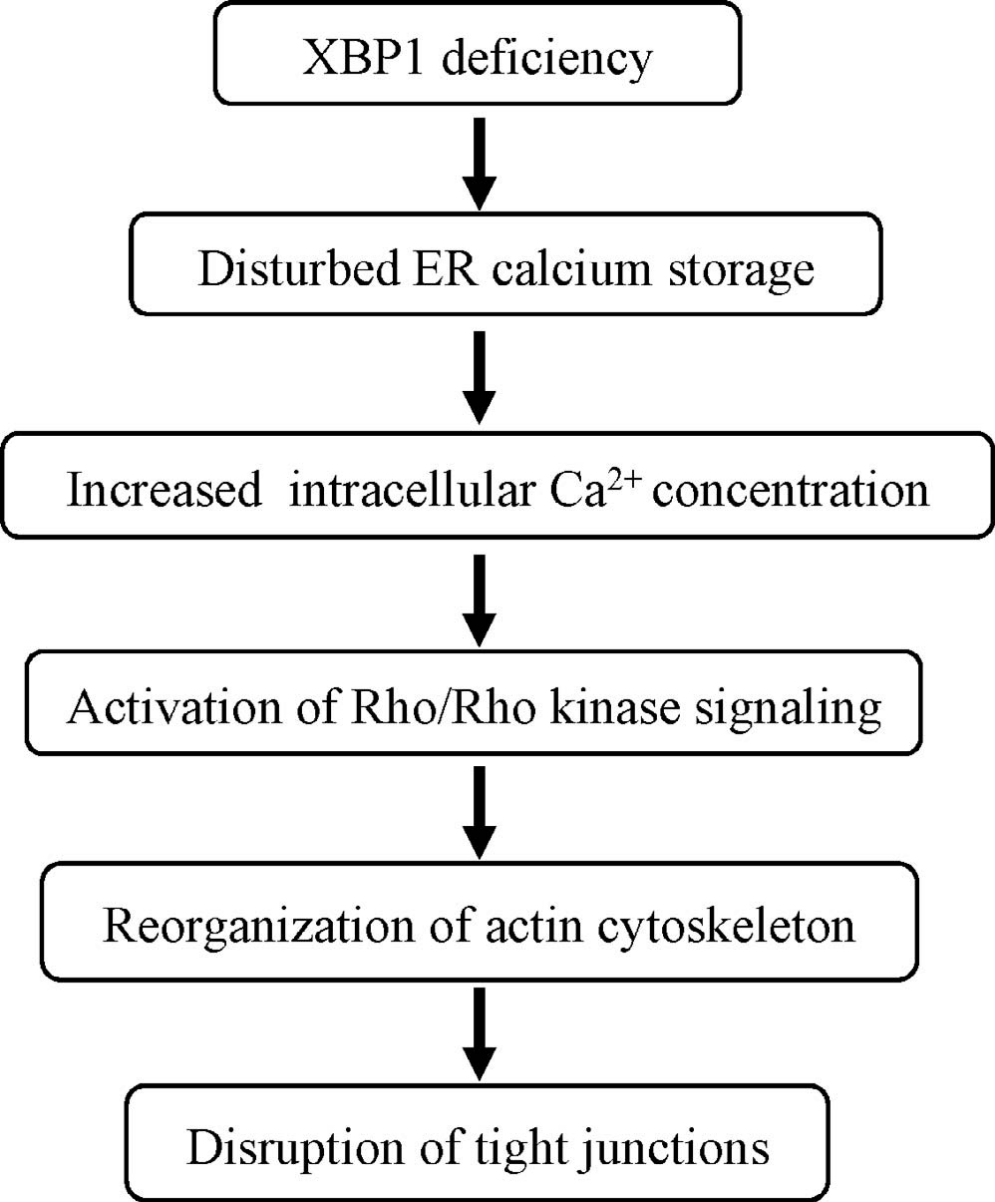
Simplified summary diagram showing potential mechanisms of XBP1 regulation of RPE tight junctions.

Dysfunction of the BRB plays an important role in the pathophysiology of several blinding diseases such as DR and AMD.^31–33^ Reduced expression and/or dislocation of the RPE tight junction proteins, resulting in increased RPE permeability, disrupts the integrity of the outer BRB, and causes disturbed function of adjacent retinal neurons,^34^ yet the regulatory mechanisms of RPE tight junctions are not fully understood. In this study, we demonstrated that depletion of calcium from the ER by thapsigargin led to interruptions in the circumferential continuity of RPE tight junctions. Similarly, inhibition of XBP1 activation increased intracellular Ca^2+^ concentration, resulting in upregulation of RhoA expression, redistribution of actin filaments, and both impairment of morphologic integrity as well as subcellular distribution of tight junctions. Inhibition of RhoA/Rho kinase pathway or overexpression of XBP1s protects RPE from derangement of tight junctions caused by XBP1 inhibition. These results suggest a protective role of XBP1 in maintaining RPE tight junctions possibly through regulation of calcium-dependent RhoA/Rho kinase signaling and actin cytoskeleton organization (Fig. 7).

In our study, we observed distinct effects of ER stress inducers tunicamycin and thapsigargin on tight junction proteins in ARPE-19 cells. These results are opposite to a recent report by Yoshikawa and colleagues,^9^ which shows that inducing ER stress by tunicamycin or thapsigargin increases the expression of ZO-1, occludin, and claudin-1. While the exact cause of the discrepancies remains unclear, we speculate that it is largely associated with the differences in cell status and culture conditions. In the Yoshikawa study,^9^ proliferating cells cultured in medium with high concentration of serum were used. In contrast, in our study the cells were made quiescent and differentiated by culture in serum-free or highly reduced serum-containing media, which perhaps would better recapitulate the in vivo situation.^35^ To confirm the results from ARPE-19 cells, we employed MRPE cells in early passage. As expected, the latter cells show greater resistance to ER stress inducers compared to ARPE-19 cells. Interestingly, in both ARPE-19 and MRPE cells inhibition of XBP1 activation by a sublethal dose of 4μ8C (Supplementary Figures) results in disruption of tight junctions and disorganization of actin cytoskeleton. Furthermore, our in vivo study shows that XBP1 deletion in RPE cells leads to impaired tight junction formation. Conversely, overexpression of active XBP1 increases ZO-1 protein production and prevents 4μ8C-induced tight junction damage. Together, these results provide strong evidence suggesting a protecting role of XBP1 in maintaining the RPE tight junctions.

Polarity is an important feature of differentiated epithelial cells and is vital for the formation of epithelial barrier. Polarized epithelial cells show apically located tight junctions and complex of perijunctional cytoskeleton composed of actin filaments with the appearance of a thick circumferential belt. The well-controlled actin rearrangement and interaction with junctional proteins warrant the integrity of RPE barrier function and the regulation of these processes involves the small Rho GTPase.^36,37^ In RPE cells, activation of Rho kinase has been shown to induce aberrant cytoskeletal reorganization resulting in disrupted tight junctions or adherens junctions.^38,39^ Similarly, oxidative damage of RPE cells by cigarette smoke extract also resulted in F-actin rearrangement accompanied by defects in both adherens and tight junctions.^12^ Herein, we found that XBP1 inhibition resulted in disruption of tight junctions due to dislocation of ZO-1 and occludin. These proteins appeared to be closely associated with actin filaments that were redistributed from the apical to basal compartments of polarized RPE cells. Further, treatment of cells with thapsigargin or XBP1 inhibitor results in upregulation of RhoA. Suppression of Rho kinase activity largely prevented actin cytoskeletal rearrangement and tight junction damage induced by thapsigargin or XBP1 inhibition. These findings suggest that suppression of XBP1 results in disruption of RPE tight junctions likely through Rho/Rho kinase-dependent cytoskeletal disorganization and cell depolarization.

Another interesting observation in our study is that two ER stress inducers, tunicamycin and thapsigargin, demonstrate distinct effects on tight junction regulation, which, we suspect, is attributed to their different mechanisms of action. Thapsigargin as a SERCA inhibitor is known to deplete the ER calcium storage and consequently raises intracellular calcium concentration. In line with our findings in RPE cells, thapsigargin treatment perturbs tight junction formation in polarized kidney epithelial cells.^40^ This effect is believed to be mediated by alteration of intracellular calcium concentration.^40,41^ Mechanistic studies have shown that disturbance in calcium homeostasis activates the Rho/Rho kinase signaling pathways, resulting in disassociation and reassembly of tight junction complexes.^42–44^ Inhibition of Ca^2+^ release by BAPTA-AM or inositol 1,4,5-triphosphate receptor (IP3R)-antagonist successfully prevents ethanol induced disruption of intestinal epithelial tight junctions.^45^ In agreement with thapsigargin's molecular mechanism, we found that blocking XBP1 activation resulted in elevated intracellular Ca^2+^ concentration in RPE cells. In line with these results, Akiyama et al.^20^ reported that lack of XBP1 gene increased the intracellular calcium concentration in pancreatic α cells. Conversely, activation of XBP1 downregulates the ryanodine calcium channel and prevents the accumulation of free calcium in the cytosol during ER stress.^46^ Ryanodine receptors (RyRs) are major ion channel proteins responsible for ER calcium release and their dysregulation is implicated in neurodengerative diseases such as Alzheimer's disease. Reducing RyR activity protects again amyloid-β–induced eye degeneration.^46^ These findings strongly support a role of XBP1 in regulation of calcium homeostasis and maintaining tight junction integrity in RPE cells, whether this effect is mediated by regulation of RyRs remain elusive.

Besides XBP1 mRNA splicing, IRE1 is also responsible for the cleavage of other ER-located mRNAs leading to their degradation through regulated IRE1-dependent decay (RIDD).^47^ Mutation of IRE1 gene results in a greater alteration of ER homeostasis than mutation of XBP1, suggesting that RIDD activity is also required to maintain the ER homeostasis.^48^ A recent study shows that inhibition of IRE1 by 4μ8C not only suppresses XBP1 splicing but also diminishes cleavage of miR-150, resulting in decreased expression of aSMA in myofibroblast and reduced fibrosis in vivo.^49^ In addition, a large number of RIDD substrates have been recently reported and are implicated critically in maintaining ER homeostasis.^47^ Whether other RIDD substrates affected by 4μ8C contribute to its effects on RPE tight junctions is yet to be elucidated.

Lastly, we show that in vivo XBP1-deficient RPE expresses enhanced VEGF coincident with impaired tight junctions. Increased proinflammatory cytokines including VEGF have been shown to be potent inducers of tight junction damage.^27^ Loss of XBP1 in intestinal epithelial cells results in spontaneous inflammation and pathologic changes of inflammatory bowel disease.^50^ In retinal cells, activation of XBP1 suppresses nuclear factor kappa-B (NF-κB) signaling and endothelial inflammation.^19^ These results suggest that enhanced VEGF expression may contribute to pathology of RPE tight junctions in XBP1-deficient RPE cells. Taken together, our study demonstrates a critical role of XBP1 in modulation of RPE tight junctions likely through mechanisms involving calcium homeostasis, Rho/Rho kinase signaling, actin cytoskeletal organization, and inflammation. Future studies will identify the specific molecular targets in regulation of these signaling pathways and explore potential implications of other UPR pathways, such as ATF6 or PERK/ATF4/CHOP, in ER stress-associated RPE barrier disruption or dysfunction and in relevant diseases such as diabetic retinopathy.

## Supplementary Material

Supplement 1

Supplement 2
