# Supplementary material for: The Role of IRE-XBP1 Pathway in Regulation of Retinal Pigment Epithelium Tight Junctions
Source: Invest Ophthalmol Vis Sci. 2016 Oct;57(13):5244–52. doi: 10.1167/iovs.16-19232 (PMC5054729; doi:10.1167/iovs.16-19232)
Supplement: Supplement 1 [file iovs-57-11-06_s01.pdf]

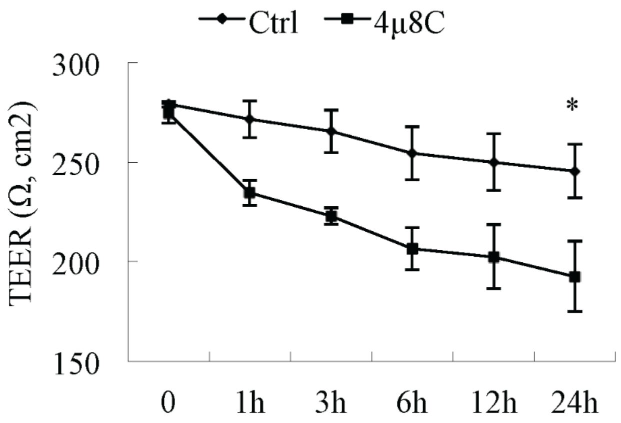

Suppl. Fig. 1. 4μ8C reduces transepithelial electrical resistance (TEER) in ARPE-19 cells. ARPE-19 cells were seeded in transwell inserts, grown to confluent, and cultured in serum-free medium for 3 weeks to induce polarization**.** Cells were exposed to 25 μM of 4μ8C for up to 24 h. TEER was measured with an EVOMX voltohmmeter (World Precision Instruments, Sarasota, FL, USA) at 1, 3, 6, 12, and 24 h after 4μ8C treatment. Data are expressed as mean ± SD from 2 independent experiments. * $p<0.05$ vs. control, ANOVA.

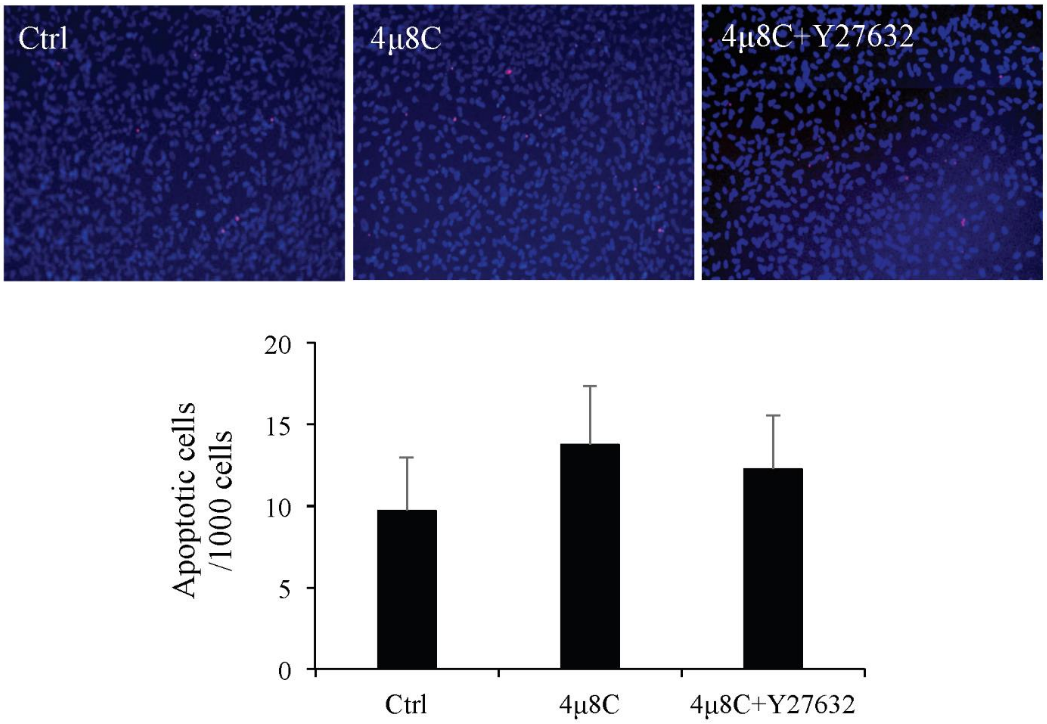

Suppl. Fig. 2. Effect of 4μ8C and Y27632 on apoptosis of ARPE-19 cells. Differentiated ARPE-19 cells were treated with 25 μM 4μ8C in the present or absence of 1 μM of Y27632 for 24 h. Cell apoptosis was examined with In Situ Cell Death Detection Kit (Roche). Data are expressed as mean ± SD from 3 independent experiments. No significant difference was identified by ANOVA analysis.

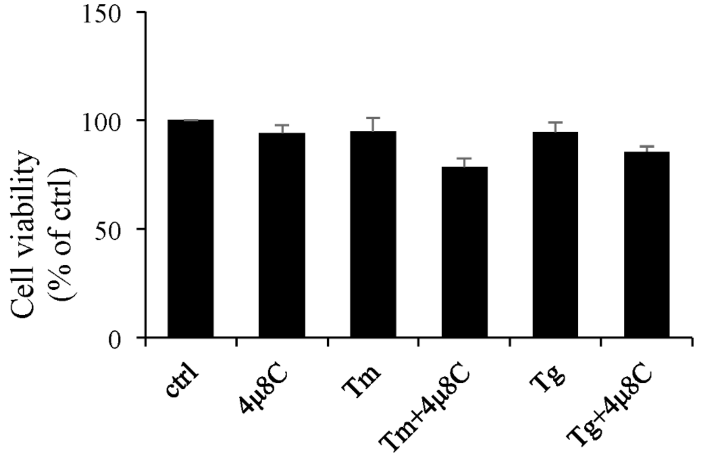

Suppl. Fig. 3. Cell viability in ARPE-19 cells. Differentiated ARPE-19 were pretreated with 25 μM 4μ8C followed by Tm (1 μg/ml) or Tg (1 μM) treatment for 24 h. Cell viability was evaluated using MTT Cell Proliferation/Viability Assay MTT kit (R&D Systems). Data are expressed as mean ± SD from 2 independent experiments. No significant difference was identified by ANOVA analysis.
