# Supplementary material for: The Role of IRE-XBP1 Pathway in Regulation of Retinal Pigment Epithelium Tight Junctions
Source: Invest Ophthalmol Vis Sci. 2016 Oct;57(13):5244–52. doi: 10.1167/iovs.16-19232 (PMC5054729; doi:10.1167/iovs.16-19232)
Supplement: Supplement 2 [file iovs-57-11-06_s02.pdf]

**Supplemental Materials and Methods**

Basal medium for “low” calcium medium formulation for monkey RPE cells:
Calcium-free DMEM (Gibco) / KBM gold (Lonza #195130), 1:1

Basal medium for “high” calcium medium formulation:
DMEM with HEPES (Sigma)/ Calcium-free DMEM (Gibco) / KBM gold (Lonza), 5:4:9

Supplemental components in both media:
TAPSO (Sigma-Aldrich)
Bovine Calf Serum (Lonza)
Vitamin E acetate (Sigma)
Linoleic acid (Cayman Chemical), complexed with Bovine Serum Albumin (Sigma)
Transferrin (Sigma)
LONG $R^3$IGF-1 (Sigma)
Oxalacetic Acid (Sigma)
Sodium pyruvate (Sigma)
Glucose (Sigma)
Fructose (Sigma)
Glucosamine (Sigma)
Sodium glucuronate (Sigma)
Gluconolactone (Sigma)
Lactose (Sigma)
Galactose (Sigma)
Fucose (Sigma)
Non-essential amino acids (Sigma)
Taurine (Sigma)
Heparin (Sigma)
Thiamine (Santa Cruz Biotechnology)
Hypoxanthine (Sigma)
Uridine (Sigma)

Alanyl-glutamine (Sigma)

Hydrocortisone (Sigma), complexed with hyroxypropyl-β-cyclodextrin (Sigma)

Triiodothyronine (Sigma)

Carnitine tartrate (LKT Labs)

Glutathione SH (Sigma)

Progestrone, soluble (Sigma)

Retinyl acetate, soluble (Sigma)

Ascorbic acid $PO_4$ (Wako Chemical)

Putrescine (Sigma)

Thioglycerol (Sigma)

Calcium chloride (Sigma)

Extract of bovine retinas (Animal Technologies; final added protein concentrations: 10 μg/ml [low calcium]; 5 μg/ml [high calcium])
